# Comprehensive functional and anatomic assessment of myocardial bridging: Unlocking the Gordian Knot

**DOI:** 10.3389/fcvm.2022.970422

**Published:** 2022-11-08

**Authors:** Giuseppe Ciliberti, Renzo Laborante, Marco Di Francesco, Attilio Restivo, Gaetano Rizzo, Mattia Galli, Francesco Canonico, Andrea Zito, Giuseppe Princi, Rocco Vergallo, Antonio Maria Leone, Francesco Burzotta, Carlo Trani, Vincenzo Palmieri, Paolo Zeppilli, Filippo Crea, Domenico D’Amario

**Affiliations:** ^1^Department of Cardiovascular and Thoracic Sciences, Catholic University of the Sacred Heart, Rome, Italy; ^2^Maria Cecilia Hospital, Gruppo Villa Maria (GVM) Care and Research, Cotignola, Italy; ^3^Department of Cardiovascular and Thoracic Sciences, Fondazione Policlinico Universitario Agostino Gemelli Istituto di Ricovero e Cura a Carattere Scientifico (IRCCS), Rome, Italy; ^4^Sports Medicine Unit, Fondazione Policlinico Universitario Agostino Gemelli Istituto di Ricovero e Cura a Carattere Scientifico (IRCCS), Rome, Italy

**Keywords:** myocardial bridging, myocardial ischemia, invasive intracoronary assessment, intracoronary physiology, intracoronary imaging, non-invasive tests, tailored therapy

## Abstract

Myocardial bridging (MB) is the most frequent congenital coronary anomaly in which a segment of an epicardial coronary artery takes a tunneled course under a bridge of the myocardium. This segment is compressed during systole, resulting in the so-called “milking effect” at coronary angiography. As coronary blood flow occurs primarily during diastole, the clinical relevance of MB is heterogeneous, being usually considered an asymptomatic bystander. However, many studies have suggested its association with myocardial ischemia, anginal symptoms, and adverse cardiac events. The advent of contemporary non-invasive and invasive imaging modalities and the standardization of intracoronary functional assessment tools have remarkably improved our understanding of MB-related ischemia, suggesting the role of atherosclerotic lesions proximal to MB, vasomotor disorders and microvascular dysfunction as possible pathophysiological substrates. The aim of this review is to provide a contemporary overview of the pathophysiology and of the non-invasive and invasive assessment of MB, in the attempt to implement a case-by-case therapeutic approach according to the specific endotype of MB-related ischemia.

## Introduction

Myocardial Bridging (MB) is the most common inborn coronary artery variant in which a segment of an epicardial coronary artery, most frequently (70–98%) the left anterior descending (LAD) coronary artery, takes an intramural course under a bridge of myocardium (1).

Coronary angiography (CA) has been typically considered the gold standard for the diagnosis of MB, detecting the “milking effect” induced by the systolic compression of the intramural artery in addition to its delayed diastolic relaxation (2–4). Nevertheless, CA may underestimate the presence of MB, whose incidence depends on the modality used to identify the tunneled segment. Indeed, MB has been documented from 5 to 8% of invasive angiographic series, but from 18 to 25% or 30 to 55% when using coronary computed tomography angiography (CCTA) or autopsy reports, respectively (5).

As coronary blood flow occurs primarily during diastole, the clinical relevance of MB is still a matter of debate, being typically considered as an innocent bystander (6). However, several studies documented that patients with MB experienced a high burden of anginal symptoms, angina-equivalents (i.e., dyspnea) and, less frequently, palpitations and/or ventricular arrhythmias (7). Although this anomaly is present at birth, the onset of symptoms usually does not occur before the third decade of age. Several factors, such as concomitant coronary artery disease (CAD), tachycardia, and the rise in left ventricular (LV) pressures, usually associated with aging, diastolic dysfunction and LV hypertrophy, may worsen the supply-demand mismatch imposed by MB and unmask or exacerbate the hemodynamic impact of MB (8, 9).

Interestingly, MB patients may also present with acute coronary syndrome (ACS), as a result of coronary artery spasm (CAS), coronary artery dissection, or coronary artery thrombosis (10).

**FIGURE 1:**
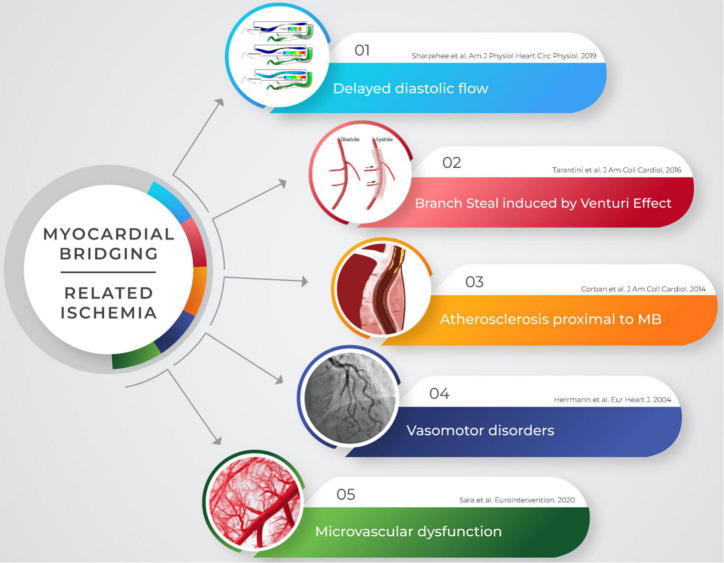
Myocardial ischemia is not purely related to vessel systolic compression in patients with MB. Several mechanisms may account for the occurrence of symptoms and may be detected though invasive and non-invasive modalities. MB, myocardial bridging.

Nowadays, a growing body of evidence suggests a direct association between MB and myocardial ischemia. Moreover, MB has been recently recognized as a cause of ischemia with non-obstructive coronary artery disease (INOCA) (11), and several mechanisms have been described as possible pathophysiological substrates of MB-related ischemia (8, 10, 12, 13; Figure 1).

Nevertheless, these multiple mechanisms cannot be unmasked by a single diagnostic modality (Table 1).

**TABLE 1:** Invasive and non-invasive diagnostic modalities for the detection, anatomic and functional assessment of MB.

Imaging modality	Diagnostic sign	Prevalence of MB (%)	Limitations	Strong points
CA	Milking effect	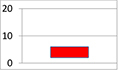	- Invasive - No functional assessment - Contrast agent - Radiation	- Anatomic assessment - Relatively fast and simple procedure
				
FFR	Pressure drop (<0.75 or 0.8)	N/A	- Invasive - Contrast agent - Radiation Longer procedural time - Pharmacotherapy side effects (adenosine)	- Functional gold standard for CAD - Hemodynamic assessment of MB
Intracoronary Doppler	Fingertip phenomenon	N/A	- Invasive - Value in guiding treatment no standardized - Contrast agent - Radiation - Longer procedural time - Pharmacotherapy side effects (adenosine, dobutamine)	- Lesion-specific sign - Functional assessment of coronary lesions - Assessment of microvascular disease or endothelial dysfunction
iFR	Pressure drop (≤0.85)	N/A	- Invasive - Contrast agent Radiation - Longer procedural time	- Diastolic specific-index - Functional assessment of CAD - Hemodynamic assessment of MB
IVUS	Half-moon sign	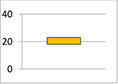	- Invasive - No functional assessment - Contrast agent - Radiation - Longer procedural time - Operator-dependent variability in pullback velocity may influence detection and morphological assessment of MB	- Lesion specific sign - Morphological assessment of proximal plaque - Quantification of persistence of arterial compression during diastole Guide to PCI
				
OCT	Heterogeneous fusiform band with intermediate-intensity signal surrounding the vessel adventitia	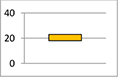	- Invasive - No functional assessment - Contrast agent - Radiation - Longer procedural time - Operator-dependent variability in pullback velocity influencing detection and morphological assessment of MB	- Lesion specific sign - Morphological assessment of proximal plaque - Guide to PCI
				
CCTA	Intramural coronary artery	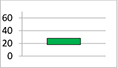	- Radiation - Contrast agent - Pharmacotherapy side effects (nitrates, BBs) - No functional assessment	- High-sensitivity - Non-invasive - High spatial resolution - Well-standardized - Concomitant detection of CAD
				
FFR-CCTA	Intramural coronary artery	N/A	- Radiation - Contrast agent - Pharmacotherapy side effects (nitrates, beta-blockers) - Longer procedural time - Value in guiding treatment no standardized	- Functional assessment - High-sensitivity - Non-invasive - High spatial resolution - Concomitant detection of CAD
Echocardiogram	Septal buckling with apical sparing or lower septal longitudinal strain with exercise	N/A	- Inability to distinguish between CAD and MB - No anatomic assessment - Operator-dependent variability - Value in guiding diagnosis and treatment not standardized	- Non-invasive - No radiation - No contrast exposure - Wall motion assessment - Fast and rapidly available
CMRI	Intramural coronary artery	N/A	- Contrast agent - Value in guiding diagnosis and treatment not standardized	- Non-invasive - No radiation - Anatomic assessment - Functional assessment - Wall motion assessment

BBs, beta-blockers; CA, coronary angiography; CAD, coronary artery disease; CCTA, coronary computed tomography; CMRI, cardiac magnetic resonance imaging; FFR, fractional flow reserve; iFR, instantaneous wave-free ratio; IVUS, intravascular ultrasound; MB, myocardial bridging; OCT, optical coherence tomography; N/A, not applicable; PCI, percutaneous coronary intervention.

The aim of our review is to show the role of different diagnostic strategies (both non-invasive and invasive) in the anatomical and functional assessment of MB. A comprehensive approach might play a key role in implementing personalized medical or invasive therapeutic strategies, ultimately yielding a benefit on symptoms and reducing the occurrence of adverse cardiac events (13, 14).

## Mechanisms of myocardial ischemia and clinical relevance in patients with myocardial bridging

The systolic phase of the myocardial cycle is only marginally involved in the myocardial perfusion (∼15%) (1). Nonetheless, an increasing body of evidence supports the involvement of different mechanisms of ischemia in patients with MB: delayed early diastolic artery relaxation, development of atherosclerotic stenosis proximal to MB, functional disorders of the coronary circulation (i.e., impaired endothelium-dependent vasodilatation and microvascular dysfunction) and the “branch steal” phenomenon.

The assumption that MB affects coronary blood flow during systole has been overcome since the diastolic lumen gain may be late and incomplete once the systolic compression of the tunneled artery ends (15–18). In 1993, Erbel et al. were the first to describe the delayed early diastolic artery relaxation within the tunneled segment through angiography and IVUS (19). Subsequently, other IVUS studies confirmed the same phenomenon, showing the concordance between delayed diastolic relaxation, increased intracoronary Doppler flow velocity, and ischemic symptoms and signs (20). Furthermore, stress-echocardiography (SE) studies also suggested that slow and incomplete MB decompression may underlie stress-induced ischemia in MB patients (18).

Myocardial bridging-related ischemia is not to be exclusively sought in the systo-diastolic hemodynamic modifications imposed by MB, but also in the anatomical and/or functional anomalies of the coronary circulation that may coexist or be favored by the MB itself. In this regard, an association between MB and CAD has long been described (8, 13). A local systolic retrograde flow phenomenon proximal to MB has been detected, predisposing to the development of atherosclerosis (21). Furthermore, the compression-relaxation of the intramural segment induces changes in wall shear stress (WSS), producing an area of low WSS proximal to MB (21). Low WSS induces the release of inflammatory mediators and endothelial vasoactive agents, whose levels were found to be significantly higher in the proximal segment compared with the intramural segment (2, 22). Using a parametric finite element model, Nikoliæ et al. found a correlation between the position of plaque near MB with WSS and oscillatory shear index. This finding reinforces the notion that plaque progression may be favored by hemodynamic disturbances provoked by MB (23).

On the contrary, the intramural tract is typically spared from atherosclerosis, probably because of the “separation” of the tunneled segment from epicardial adipose tissue and its pro-inflammatory signals (i.e., cytokines and adipokines) (24–26) as shown in preclinical models by the lack of foam cells and modified smooth muscle cells in the intramural artery (27).

The presence of atherosclerotic plaques proximal to MB is a potential cause of chronic coronary syndrome (CCS) (10, 15–17), as well as ACS due to plaque erosion/rupture or vasospasm and coronary dissection (10, 28–31).

Myocardial bridging has also been associated with impaired endothelium-dependent vasodilatation (32–36). Endothelial dysfunction, together with the hyper-reactivity of vascular smooth muscle cells, represent the two pathophysiological mechanisms implicated in the occurrence of CAS (37). Previous studies, using a provocative test with incremental acetylcholine (ACH) dose infusion, demonstrated that CAS is more common in patients with MB (32–36). Furthermore, anatomical properties of MB, such as length and percentage of systolic compression, were demonstrated to predict the occurrence of provoked LAD spasm (32, 34).

It has been proposed that the turbulent flow and changes in WSS, due to MB, may promote direct injury to the endothelium and endothelial cell apoptosis (33, 35). Moreover, the expression of endothelial nitric oxide synthase (eNOS), a marker of preserved endothelial function, is significantly lower in the MB segment than in proximal and distal segments (36). These mechanisms ultimately converge to a paradoxical response to ACH and an increased risk of CAS (36). The occurrence of CAS in MB patients may have significant clinical relevance: Nam et al. reported that patients with MB and CAS experienced a higher rate of recurrent angina and a more frequent prescription of anti-anginal medication (32). Furthermore, according to the correlation between MB and myocardial infarction risk (38), CAS may also represent a mechanism underlying the development of ACS (39).

Endothelial dysfunction is, however, not confined to the epicardial coronary artery but may extend to the coronary microcirculation (11). Indeed, it is not unlikely that impaired endothelium-dependent vasodilatation affects also distal arterioles, resulting in microvascular spasm as well as spasm of the epicardial tract strictly close to MB (40).

Nowadays, little data is available on the link between MB and coronary microvascular dysfunction (CMD). CMD is prevalent across several cardiovascular conditions, emerging as an increasing cause of INOCA (41, 42). Two different endotypes of microvascular dysfunction currently exists: structural microvascular remodeling and functional arteriolar dysregulation (11). The former is caused by an increase in wall to lumen ratio and a loss of myocardial capillary density, and it is characterized by an impaired endothelium-independent vasodilatation, represented by a reduced coronary flow reserve (CFR) and an increased index of microcirculatory resistance (IMR) (11). The latter is caused by endothelial dysfunction of medium and large size arterioles, with a pathological response to ACH test (impaired endothelium-dependent vasodilatation) (11).

Only two studies investigated the presence of CMD in patients with MB (40, 43). Among patients with chest pain and non-obstructive CAD, Sara et al. showed that those with angiographic evidence of MB had a higher frequency of microvascular endothelial dysfunction compared to patients without MB (57.7 vs. 51.0%, *p* = 0.075). However, this reached statistical significance among patients aged ≤ 50 years (57.3 vs. 44.2%, *p* = 0.010) (40). The second study showed a high rate of CMD (22.1%) among patients with persistent angina, non-obstructive CAD and angiographic evidence of MB (43). On the basis of these results, CMD may represent an additional mechanism of ischemia in patients with MB. Therefore, a comprehensive invasive assessment of MB patients that includes the evaluation of coronary microcirculation (i.e., IMR and CFR) might provide additional information. However, further data are needed to suspect a more marked involvement of microvascular dysfunction in these patients.

Lastly, the “branch steal effect” is an additional ischemic mechanism in MB patients: the crossing of blood through the constrict segment in the end systole/early diastole leads to an increase in diastolic flow velocity (“Venturi effect”) that results in a depressurization at the ostium of side branches within the MB (44, 45). This phenomenon is more evident in the case of septal perforator arteries of the LAD coronary artery (7). Interestingly, Lin et al. proposed this mechanism to explain the echocardiographic finding of “septal wall motion abnormality” during dobutamine stress test as sign of focal ischemia. Conversely, the recovery of perfusion pressure distal to the LAD was not associated with ischemic signs (i.e., echocardiographic finding of “apical sparing”) (44).

In summary, several mechanisms suggest the ischemic relevance of MB: exertion-induced angina may occur for a delay in early diastolic artery relaxation, “branch steal,” hemodynamically significant proximal CAD and CMD. On the other hand, epicardial spasm, coronary artery dissection, and coronary artery thrombosis may underlie the development of ACS.

## Non-invasive anatomical and functional assessment

Although CA may detect the presence of MB, there are specific patient-dependent and procedure-related complications that are inherent to the invasive procedure (46). Some non-invasive imaging modalities may be useful to detect MB and to investigate myocardial ischemia in MB patients, since this cohort of patients is typically younger, has a lower rate of cardiovascular risk factors and, therefore, a low pre-test probability of CAD (47).

Among non-invasive imaging tests, CCTA plays a prominent role. Its strength is represented by a high spatial resolution, accounting for a higher detection of MB compared with CA (48). CCTA enables an accurate evaluation of the vessel wall and lumen, as well as the surrounding myocardium, allowing to classify MB of the LAD coronary artery in terms of depth and length, suitable only for MB located in the LAD coronary artery (49). In this regard, MB is classifiable into superficial (<2 mm), deep (≥2 mm), and very deep (≥5 mm) according to the depth; and into short (<25 mm) or long (≥25 mm) according to the length of the tunneled segment (49, 50). Kim et al. proposed an alternative classification of LAD MB into three types: the type I, characterized by a partial muscular encasement; the type II, characterized by a full encasement of the vessel by myocardium but without measurable overlying myocardium; the type III, characterized by a measurable overlying myocardium > 0.7 mm (48).

These anatomical evaluations have relevant clinical, prognostic and therapeutical implications: indeed, the deeper variant is more commonly associated with symptoms and adverse cardiac events; moreover, CCTA may be helpful to guide the choice of surgical treatment (myotomy vs. CABG) (13, 51).

The pivotal limitation of CCTA is the low temporal resolution, making difficult to assess the hemodynamic significance of MB. Moreover, the use of a “cocktail” of drugs before scanning, such as beta-blockers and nitroglycerin that prolong diastolic time and induce vasodilation, respectively, results in an underestimation of the impairment in blood flow imposed by MB (13).

Among non-invasive tests, echocardiogram plays a role in the functional assessment of MB patients. Lin et al. described an echocardiographic finding characterized by a focal abnormality in the end-systolic to early-diastolic septal wall motion with apical sparing (44). Eighteen patients with angina and this sign were prospectively enrolled for invasive assessment (CA, IVUS, and intracoronary pressure measurements): all these patients were found to have MB of the LAD coronary artery and an abnormal diastolic-fractional flow reserve (dFFR ≤ 0.75) (44). In addition, patients with hemodynamically significant LAD MB present lower septal longitudinal strain compared to controls during stress echocardiographic strain imaging (52). “Septal buckling with apical sparing” has a diagnostic accuracy similar to SE in identifying significant CAD through a new wall motion abnormality (52). However, even though SE is a well-established diagnostic tool in detecting myocardial ischemia, its key limitation is related to the impossibility to understand if the wall motion abnormalities are attributable to MB, CAD or other pathological conditions, if an anatomical assessment is not performed.

The evaluation of coronary flow velocity reserve (CFVR) measured by transthoracic Doppler echocardiography (TTDE) has been found to represent another modality to hemodynamically assess LAD MB. In this regard, Aleksandric et al. showed that non-invasive CFVR-TTDE during dobutamine infusion was a predictor of functional significant MB, found to be more accurate than CFVR during adenosine infusion (18, 53). A cut-off value of ≤2.1 was found to have the best accuracy for identifying MB associated with stress-induced myocardial ischemia with a sensitivity (Sn), specificity (Sp), positive (PPV), and negative predictive value (NPV) of 96, 95, 88, and 98%, respectively (area under curve AUC 0.986) (53). Inotropic stimulation with dobutamine, compared to vasodilatation with adenosine, provides better significance of MB in relation to stress-induced myocardial ischemia in the invasive setting too (4). Interestingly, the cut-off value of ≤2.1 is similar to the cut-off value for TTDE-CFVR during adenosine as well as during dobutamine provocation in the functional assessment of fixed coronary stenosis (Sn 92 vs. 92%; Sp 90 vs. 86%; PPV 85 vs. 73%; NPV 95 vs. 96%) (54, 55).

Among non-invasive diagnostic modalities, cardiovascular magnetic resonance imaging (CMRI), single-photon emission computed tomography (SPECT), and positron emission tomography (PET) provide the quantification of myocardial blood flow at rest and during induced-hyperemia, representing possible tools in the assessment of inducible-ischemia and CMD in MB patients (41).

However, little data supported the role of these modalities in detecting ischemia in patients with MB and anginal symptoms, and there are no standardized diagnostic criteria for functional evaluation of MB with non-invasive stress testing or myocardial perfusion imaging because previous studies were based on small number of patients (56). Further studies are needed to better validate these tools.

## Invasive anatomical and functional assessment

### Intracoronary imaging

If we consider the gap in incidence rate between CA and other imaging modalities, routine CA is not completely sensitive for the detection of MB (5). The use of intracoronary vasodilators (i.e., nitrates) can increase, through a reflex rise of the adrenergic drive, the systolic narrowing of the tunneled artery and the angiographic sensitivity in diagnosing MB (57). Nevertheless, in patients with thin MB, the “milking effect” may be missed and invasive intracoronary imaging techniques may be required to limit the underdiagnose of MB.

Intravascular ultrasound (IVUS) has been used throughout a multitude of studies for the morphological assessment of MB. It allows for accurate measurement of the lumen diameter and evaluation of vessel wall morphology (58). IVUS is able to detect the systolic compression of the bridged segment, but the peculiar finding is an echolucent area between the tunneled artery and the epicardial tissue, persisting throughout the cardiac cycle. This phenomenon is called “half-moon” sign and represents a muscle band overlying the tunneled segment (1). This sign is highly specific because it is detectable only in the segment with systolic compression (59). Moreover, IVUS confirms that vessel compression within MB is not exclusively a systolic event but it extends to early diastole (59). Additionally, IVUS is a useful tool for the quantitative and qualitative assessment of atherosclerosis proximal to MB. In this regard, Yamada et al. found that the percentage of arterial compression was directly related to the atherosclerotic burden located proximally to MB (60).

Similarly, optical coherence tomography (OCT) may represent an adjuvant tool in the diagnosis of MB, detecting angiographically undetectable MB (61). OCT is a light-based technique that provides *in vivo* high-resolution (∼10 μm) imaging of coronary artery (62). Although its resolution is higher than IVUS, the role of OCT in detecting MB has not been completely elucidated. Some studies proposed the main OCT features of MB, describing a heterogeneous fusiform band with sharp borders and low/intermediate-intensity signal, similar to tunica media, surrounding the vessel adventitia. Obviously, the fusiform band detected by OCT, corresponds to the “half-moon” sign using IVUS (63–67). OCT provides the detection and anatomic characterization of atherosclerotic lesions proximal to MB too (64). Furthermore, in cases of ACS and concomitant presence of MB, OCT helps in differentiating between different pathophysiological subtypes, such as plaque rupture/erosion, thrombosis or spontaneous coronary artery dissection (SCAD) (30). Finally, despite weak evidence, the use of OCT might help in guiding percutaneous revascularization, with the aim of minimizing peri- and post-procedural complications during stent implantation in a MB segment (63).

The key limitation of IVUS and OCT in the assessment of MB is related to the lack of functional and hemodynamic information on both MB and proximal atherosclerotic lesions. Furthermore, they increased procedural cost and the risk of underestimating the length of MB when rapid pullback is performed (Table 1).

In conclusion, the use of invasive anatomical assessment with imaging tools such as IVUS and OCT allows to maximize the diagnosis of MB, quantify arterial compression, characterize the coexisting proximal CAD, and identify the mechanism underlying the occurrence of ACS.

### Physiological assessment of myocardial bridging

**FIGURE 2:**
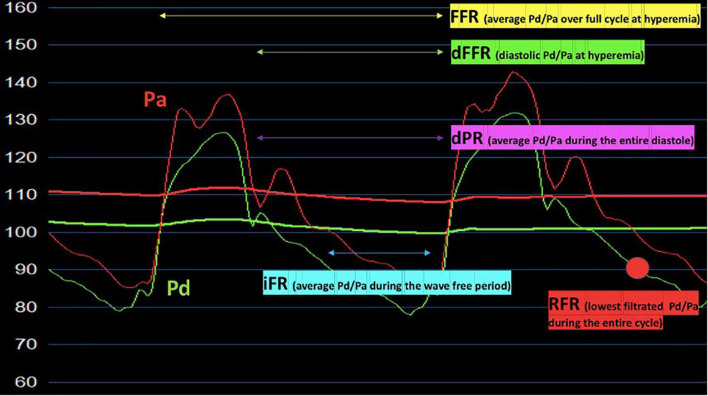
Hyperemic and non-hyperemic pressure ratios (FFR, dFFR, iFR, RFR, dPR) proposed for the invasive functional assessment of patients with myocardial bridging. Pa, aortic pressure; Pd, distal pressure; FFR, fractional flow reserve; dFFR, diastolic-fractional flow reserve; iFR, instantaneous wave-free pressure ratio; RFR, resting full-cycle ratio dPR, diastolic pressure ratio.

Functional assessment tools should be considered complementary to imaging tests. In this regard, the Doppler flow wire and pressure wire methods represent useful modalities to define the hemodynamic impact of MB (13; Figure 2).

Fractional flow reserve (FFR) is a pressure wire-based index that, through the measurement of the trans-stenotic pressure gradient during maximal hyperemia (achieved by adenosine administration), is recommended to assess the hemodynamic relevance of intermediate-grade stenosis when evidence of ischemia is not available (68, 69). To date, 0.80 is the accepted FFR threshold for defining hemodynamically relevant fixed stenosis (68, 69).

Although FFR is generally considered the gold standard for the physiological invasive assessment of atherosclerotic plaque, it may fail to identify the hemodynamic impact of dynamic coronary obstructions such as MB (14). FFR is based on the assumption that the difference between mean and diastolic pressure gradient values across the lesion is not significant: this is true for fixed stenosis, but it might not be valid for dynamic flow obstruction (7, 14). MB reduces systolic pressure gradients, as a consequence of distal pressure overshooting during myocardial contraction. This leads to an overestimation of the mean pressure (evaluated by FFR) and to an underestimation of the hemodynamic significance of MB (7). Therefore, specific diastolic functional indices have been developed to overcome the limits of FFR (13), such as dFFR which has proven to be more sensitive than conventional FFR for functional assessment of MB (70).

Moreover, as MB is a dynamic stenosis deeply influenced by the degree of extravascular compression and intra-myocardial tension, the assessment during rest may underestimate the hemodynamic relevance of a significant proportion of MBs. Dobutamine represents the drug of choice for inotropic stimulation in MB patients (18). In this regard, Escaned et al. evaluated the usefulness and safety of combining dobutamine challenge with FFR and dFFR in the presence of MB. This investigation revealed that dobutamine challenge enhanced diagnostic sensitivity of both FFR and dFFR. Interestingly, dobutamine challenge increased the discrepancy between FFR and dFFR (70). This may be explained by the decreased and negativization of systolic pressure gradient across MB segment during dobutamine provocation, leading to an artificial and paradoxical elevation in the mean pressure gradient used by traditional FFR.

The diagnostic superiority of dFFR during dobutamine provocation, compared to conventional FFR during adenosine provocation, has been recently confirmed by Aleksandric et al. using exercise-induced myocardial ischemia (4). They proposed the cut-off value of ≤0.76 for dFFR during dobutamine provocation to identify stress-induced myocardial ischemia in MB patients with a Sn, Sp, PPV and NPV of 95, 95, 90, and 98%, respectively (AUC 0.927). Curiously, this value is the same as the cut-off value used for dFFR during adenosine provocation in the functional assessment of fixed coronary stenosis (Sn 96%; Sp 100%) (71).

However, despite the promising diagnostic value, dFFR is not routinely performed in clinical practice, due to the difficulty of execution and time-consuming.

Other non-hyperemic pressure indices, including instantaneous wave-free ratio (iFR), diastolic pressure ratio (dPR), and resting full-cycle ratio (RFR) have been developed. They are obtained without the administration of vasodilators, resulting easier to perform than dFFR.

Instantaneous wave-free ratio is a pressure-derived index, recently validated for the assessment of coronary artery stenosis. It is a specific-diastolic index, calculated during a portion of diastole when vascular resistance is low and stable, the so-called “wave-free period” (72). In a prospective study by Tarantini et al., among 20 patients with angina and/or positive non-invasive stress test, absence of CAD and angiographic evidence of MB, iFR was found to be more consistent with angina and/or inducible ischemia compared with FFR. Indeed, iFR at rest was abnormal (≤0.89) in 7 patients, while no MB was hemodynamically significant according to FFR. During inotropic challenge, median FFR did not change significantly, whereas dobutamine-induced iFR resulted to be remarkable lower compared to iFR at rest (73).

Collectively, diastolic indices may identify a proportion of hemodynamically relevant MBs that are not unmasked by conventional FFR. The superiority of diastolic indices over systo-diastolic indices (i.e., FFR) in the evaluation of ischemic burden related to MB reflects the fundamental involvement of diastole in the pathophysiology of MB-related ischemia (70, 74).

Nevertheless, the evaluation of iFR depends on the assumption that maximal flow and minimal microcirculatory resistance occur during a period within diastole (the “wave-free period”), while the hemodynamic modifications imposed by MB and concomitant CAD may be not completely predictable. In this regard, two alternative non-hyperaemic pressure indices might be proposed: dPR and RFR.

Diastolic pressure ratio is the ratio between mean distal coronary pressure averaged over the entire diastolic period and the mean aortic pressure (75). Instead, RFR is calculated from the lowest value of distal pressure (Pd) and aortic pressure (Pa) over the entire cardiac cycle (76). These indices may potentially unmask significant or multiple occlusions that would be missed by an assessment dedicated only to specific periods of the cardiac cycle (75, 76).

Unfortunately, ischemic cut-off values for the above-discussed indices, at rest and during inotropic provocation, are not available for MB patients. Further studies are needed for the validation of these tools and for the definition of reliable cut-off values.

The Doppler guide wire, through selective catheterization and measuring phasic flow velocity of MB segment, reveals a typical velocity pattern termed “fingertip” phenomenon, characterized by an abrupt early-diastolic acceleration, a rapid mid-diastolic deceleration and a mid-to-late diastolic plateau (10, 13). However, the absence of the “fingertip” was found in 13% of MB patients, probably because the systolic compression sometimes is not strong enough to induce the hemodynamic changes that lead to this sign (10). Further studies are needed to confirm these results and to determine whether this phenomenon, as a marker of MB severity, predicts adverse cardiovascular events.

### Provocative test

It is well-known that endothelial dysfunction is a pathophysiological hallmark of MB-related ischemia, promoting the development of vasomotor disorders such as epicardial and microvascular CAS. In order to unmask endothelial dysfunction and CAS, three provocative tests (with ACH, ergonovine and hyperventilation) can be performed in the catheterization laboratory (77). ACH test is preferred over ergonovine and hyperventilation, because it is associated with a lower rate of complications compared to ergonovine, and it is more standardized and reproducible than hyperventilation (78). On the other hand, non-invasive provocative tests (i.e., ergonovine and hyperventilation) have been associated with significant adverse events including death, because detection and alleviation of the induced spasm may be delayed (79).

The 2019 ESC CCS guidelines support the use of intracoronary ACH test in patients with normal findings or non-obstructive lesions on coronary arteriography and clinical suspicion of CAS for the assessment of epicardial spasm (IIa recommendation) and/or microvascular spasm (IIb recommendation) (80–82). Therefore, interventional cardiologists should not refrain from performing provocative test in patients with MB and clinical picture of vasospastic angina.

Several studies investigated the relationship between CAS and MB, demonstrating that MB patients present a high rate of CAS. Furthermore, MB patients with a positive response to ACH test have a worse prognosis compared with those without spasm (10, 32–36, 83–88). However, the location of provoked spasm was not always the same: most of studies found epicardial spasm of the intramural segment. On the contrary, Saito et al. found that epicardial CAS was more frequently provoked in the proximal segment of MB (34). Moreover, heterogeneity in dose-response relationship has been noted in patients with MB and ACH-induced spasm. MB patients who responded to lower ACH doses (20 μg) have higher incidence of severe, diffuse and long (>30 mm) spasm. This results in a higher occurrence of adverse events at 12-month follow-up, compared to MB patients reacting to higher ACH doses (50 and 100 μg) (88).

Therefore, the use of ACH provocative test may be useful in the identification and quantification of a pathophysiological mechanism (impaired endothelium-dependent vasodilatation) of MB-related ischemia, with relevant prognostic and therapeutic implications.

## Therapeutic management of patients with myocardial bridging

The therapeutic management of MB patients remains a relevant challenge. Treatment options should be considered according to clinical presentation, evidence and degree of inducible ischemia, coronary and cardiac anatomy, comorbidities and patient preference (13). Nevertheless, no guidelines or expert consensus are currently available.

**FIGURE 3:**
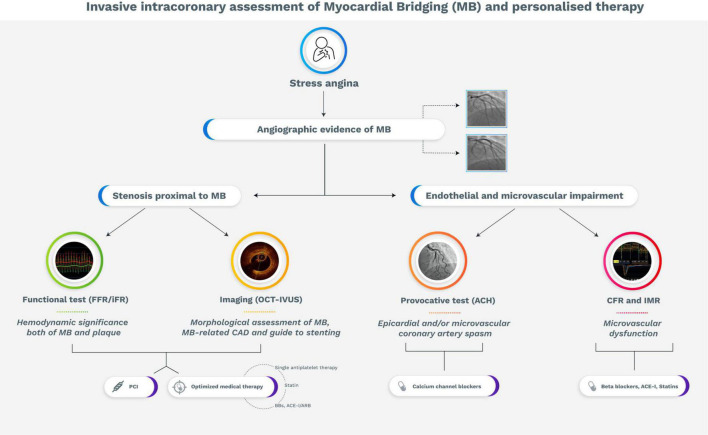
Flow diagram with proposed strategy for the management of symptomatic patients with myocardial bridging. ACE-I, Angiotensin-converting enzyme-inhibitors; Ach, acetylcholine; ARB, Angiotensin receptor blocker; BBs, beta-blockers; CAD, coronary artery disease; CFR, coronary flow reserve; FFR, fractional flow reserve; iFR, instantaneous wave-free ratio; IMR, index of microvascular resistance IVUS, intravascular ultrasound; MB, myocardial bridging; OCT, optical coherence tomography; PCI, percutaneous coronary intervention.

As discussed, the ischemic burden related to MB is supported by various pathophysiological mechanisms. In this regard, invasive and non-invasive physiological assessment, intracoronary imaging, and provocative test may unmask the dominant mechanism of myocardial ischemia, allowing to guide the treatment according to specific pathophysiological endotypes of myocardial ischemia (Figure 3).

Pharmacologic therapy is considered the first strategy for most of symptomatic MB patients (8). Beta-blockers (BBs) represent the mainstay of treatment in patients with MB and ischemic symptoms or signs of inducible ischemia. Due to their negative chronotropic effect, BBs allow to increase diastolic perfusion and diastolic filling time. In addition, they reduce compression of the tunneled vessel by their negative inotropic effect (7). Similarly, non-dihydropyridine calcium channel blockers (CCBs) may satisfy the same target, and they should be preferred in patients with contraindications to betablockers (89).

However, BBs, especially non-selective ones, may be detrimental in MB patients with concomitant CAS. In fact, blockade of the beta-2 receptors, which mediate vasodilation, increases the risk of CAS and, supposedly, adverse cardiac events (90). On the contrary, CCBs, by combining their negative ino-chronotropic effect and vasodilatory effect, may represent the treatment of choice in MB patients with concomitant vasomotor disorders unmasked by a positive ACH test (13, 91). Conversely, nitrates, although powerful vasodilators, should be avoided since they worsen systolic narrowing and symptoms through a reflex rise of the adrenergic drive.

Since MB has been associated with an increased prevalence of CAD proximal to the bridged segment, optimized medical therapy of cardiovascular risk factors should be considered once atherosclerosis is detected (13).

If symptoms persist despite maximally tolerated medical therapy, a “revascularization” strategy should be considered. The choice among percutaneous coronary intervention (PCI), coronary artery bypass graft (CABG) or supra-arterial myotomy should be guided by anatomical features of MB (i.e., length and depth), age, concomitant CAD, and patient preference.

Randomized data are lacking for the use of PCI in the treatment of MB, and this approach was historically reserved to patients with anginal symptoms refractory to maximal medical therapy (92). Any rationale for PCI in this setting would be to treat stenosis proximal to MB as well as the dynamic obstruction within the tunneled segment, aiming at protecting it from systolic compression (8). However, PCI with stenting in MB patients may lead to a high rate of complications such as in-stent restenosis (ISR), very late stent thrombosis, and coronary perforation, due to the sustained stress over time within the intramural tract. These complications were described mainly in studies using bare-metal stent (BMS) and first-generation drug eluting stent (DES) (8, 63, 93–96). Probably, the use of second-generation DES or future bioabsorbable scaffolds may potentially limit these complications (13). Furthermore, the use of intracoronary imaging tools may provide accurate information about MB sizing, guiding the percutaneous treatment. In this regard, a recent retrospective study showed that the guidance of OCT limited the incidence of perforation and ISR in the MB segment covered with DES (63).

Given the MB stenting-related complications, surgery is an effective therapeutic alternative for symptomatic MB refractory to maximally tolerated medical therapy. Myotomy is the treatment of choice for MB with favorable anatomy (i.e., non-tortuous artery, short and superficial intra-myocardial course), especially in pediatric population (97). Conversely, myotomy presents a high rate of failure in adult patients, probably because abnormal flow related to MB causes damage to coronary circulation (i.e., endothelial dysfunction), persisting and impairing coronary flow after surgical intervention (5). CABG is superior to myotomy in case of complex anatomy (i.e., deep and/or long MB) and adult patients (51).

Unfortunately, studies assessing short- and long-term effect of antianginal drugs vs. PCI or surgical treatment in MB patients are lacking. Large and prospective randomized clinical trials are needed in this regard.

## Conclusion

Myocardial bridging has long been considered an accidental finding. However, a growing body of evidence suggested that it is associated with impaired quality of life and might lead to adverse cardiac events.

Invasive intracoronary assessment, *via* imaging techniques (i.e., OCT and IVUS), or full invasive physiological evaluation (i.e., FFR, iFR, CFR, IMR), together with the utilization of provocative test (i.e., ACH test), improved the ability to evaluate the hemodynamic relevance of MB, to understand the underlying pathophysiological mechanisms, leading to an optimization of the therapy according to each specific endotypes.

Nevertheless, the uncertainties we are currently facing in the diagnosis, characterization, and treatment of patients presenting with MB and evidence of ischemia are remarkable, and related, at least in part, to the skepticism and the paucity of evidence that support the clinical and prognostic relevance of MB in the setting of CCS and ACS. Therefore, there is an unmet need in defining unequivocally the diagnostic tools and the related cut-offs to be used, guiding the therapeutic management of patients with MB. The present review, systematically addressing the current shreds of evidence in the field, set the stage for considering MB as a novel and interesting therapeutic target, aiming to launch in the near future reliable and compelling efficacy endpoints to be utilized in proof-of-concept clinical trials.

## Author contributions

RL and GC had a leading role in writing the manuscript. DD’A had a leading role in manuscript revision. FCr, PZ, AR, GR, MD, MG, AZ, FCa, RV, GP, AL, FB, CT, and VP had a supporting role in manuscript revision. All authors have read and agreed to the content of the manuscript.
